# Impact of bystander cardiopulmonary resuscitation on neurological outcomes in patients undergoing veno-arterial extracorporeal membrane oxygenation

**DOI:** 10.1186/s12245-023-00485-1

**Published:** 2023-02-17

**Authors:** Ryosuke Shimai, Shohei Ouchi, Tetsuro Miyazaki, Koji Hirabayashi, Hiroshi Abe, Kosuke Yabe, Midori Kakihara, Masaaki Maki, Hiroyuki Isogai, Takeshi Wada, Dai Ozaki, Yuki Yasuda, Fuminori Odagiri, Kazuhisa Takamura, Kenji Yaginuma, Ken Yokoyama, Takashi Tokano, Tohru Minamino

**Affiliations:** 1 Department of Cardiology, Juntendo University Urayasu Hospital, 2-1-1 Tomioka, Urayasu-shi, Chiba, Japan; 2 Department of Cardiology, Juntendo Tokyo Koto Geriatric Medical Center, Tokyo, Japan; 3 Department of Cardiovascular Biology and Medicine, Graduate School of Medicine, Juntendo University, Tokyo, Japan

**Keywords:** Emergency cardiovascular care, Out-of-hospital cardiac arrest, The Glasgow-Pittsburgh Cerebral Performance and Overall Performance Categories of Brain Function, Intensive care

## Abstract

**Background:**

Veno-arterial extracorporeal membrane oxygenation (V-A ECMO) requires a large amount of economic and human resources. The presence of bystander cardiopulmonary resuscitation (CPR) was focused on selecting appropriate V-A ECMO candidates.

**Result:**

This study retrospectively enrolled 39 patients with V-A ECMO due to out-of-hospital cardiac arrest (CA) between January 2010 and March 2019. The introduction criteria of V-A ECMO included the following: (1) < 75 years old, (2) CA on arrival, (3) < 40 min from CA to hospital arrival, (4) shockable rhythm, and (5) good activity of daily living (ADL). The prescribed introduction criteria were not met by 14 patients, but they were introduced to V-A ECMO at the discretion of their attending physicians and were also included in the analysis. Neurological prognosis at discharge was defined using The Glasgow-Pittsburgh Cerebral Performance and Overall Performance Categories of Brain Function (CPC). Patients were divided into good or poor neurological prognosis (CPC ≤ 2 or ≥ 3) groups (8 vs. 31 patients). The good prognosis group had a significantly larger number of patients who received bystander CPR (*p* = 0.04). The mean CPC at discharge was compared based on the combination with the presence of bystander CPR and all five original criteria. Patients who received bystander CPR and met all original five criteria showed significantly better CPC than patients who did not receive bystander CPR and did not meet some of the original five criteria (*p* = 0.046).

**Conclusion:**

Considering the presence of bystander CPR help in selecting the appropriate candidate of V-A ECMO among out-of-hospital CA cases.

## Background

Over 500,000 people worldwide die from in-hospital and out-of-hospital cardiac arrest (CA) annually [1]. The survival rate is only 22% for in-hospital CA and 10% or less for out-of-hospital CA and countermeasures are required [1]. Veno-arterial extracorporeal membrane oxygenation (V-A ECMO) has been reported to be useful for CA and cardiogenic shock both in-hospital and out-of-hospital [2–5]. However, V-A ECMO requires a lot of economic and human resources, thus considering the kind of patient for its application is necessary. Currently, the criteria for introducing V-A ECMO are unclear. We focused on the presence of bystander cardiopulmonary resuscitation (CPR) as a factor that can predict a better neurological prognosis in patients with V-A ECMO. The presence of bystander CPR affects the neurological prognosis in CA cases [6], but the involvement of the presence of bystander CPR in neurological prognosis in cases using V-A ECMO is unclear. The presence of bystander CPR involved in the neurological prognosis in introducing V-A ECMO at our hospital from 10 years from 2010 to 2019 were retrospectively examined.

## Methods

### Study population

This retrospective observational study included 39 patients with out-of-hospital CA who were transported to our hospital between January 1, 2010, and March 31, 2019, and was judged to have cardiogenic CA and was introduced to V-A ECMO. The guidelines implemented for V-A ECMO introduction were as follows: (1) < 75 years old, (2) CA on arrival, (3) < 40 min from CA to hospital arrival, (4) shockable rhythm, and (5) good activities of daily living (ADL). Patients with terminal illnesses were excluded. The prescribed introduction criteria were not met by 14 patients, but they were introduced to V-A ECMO at the discretion of their attending physicians and were included in the analysis.

Bystander CPR is defined as a life-saving attempt by a person who witnesses a CA, and some doctors, nurses, and paramedics are also considered bystanders. All patients were given the optimal treatment required, including coronary angiography, percutaneous coronary intervention (PCI), intra-aortic balloon pumping (IABP), continuous blood purification therapy, and targeted temperature management (TTM). Neurological prognosis at the time of discharge was retrospectively observed. The Glasgow-Pittsburgh Cerebral Performance and Overall Performance Categories (CPC) were used to define neurological prognosis: cases with CPC1 and CPC2 were defined as having a good neurological prognosis, whereas cases with CPC3 to CPC5 were defined as poor neurological prognosis.

### Statistical analysis

Continuous variables are shown as mean and standard deviation, and categorical variables are shown as real numbers and percentages. Continuous variable comparisons were made using Student’s *t*-test or Mann-Whitney *U* test. Category variables were analyzed using the chi-square test or Fisher’s exact test. Comparisons of the average of CPC was analyzed using the Tukey-Kramer test. JMP14.2 (Windows, SAS Institute, Cary, NC) was used for statistical analysis, and a *p*-value of < 0.05 was considered statistically significant.

## Results

Table 1 shows the patient background as 56.1 ± 13.1 years old, with 33 (84.5%) males. The cause of CA includes acute coronary syndrome in 19 patients (48.7%), cardiomyopathy in 14 (35.9%), arrhythmia in 4 (10.3%), and unknown in 2 (5.1%). The mean time from onset to arrival at the hospital was 30.6 ± 3.2 min. Of all patients, 35 had ventricular fibrillation or pulseless ventricular tachycardia (89.7%), 36 (92.3%) had witnesses, and 26 (66.7%) had bystander CPR. Twelve patients (30.8%) were discharged alive. Patients were divided into two groups: those with good neurological prognosis (CPC 1 or 2, 8 patients, 20.5%) and those with poor neurological prognosis (CPC 3, 4, or 5, 31 patients, 79.5%). No significant difference was found between these two groups in terms of “<75 years old,” “shockable rhythm,” and “within 40 min from CA to hospital arrival.” No significant difference was found in the presence of witnesses between the two groups; however, the rate of bystander CPR was significantly higher in the group with a favorable prognosis (*p* = 0.04). The bicarbonate level on admission in the group with a good neurological prognosis was significantly lower than those in the group with a poor neurological prognosis (13.4 ± 4.1 vs. 17.1 ± 4.1 mmol/L, *p* = 0.04). The partial pressure of carbon dioxide (PCO_2_) levels on admission in the group with good neurological prognosis were significantly lower than those in the group with poor neurological prognosis (58.3 ± 26.4 vs. 84.7 ± 27.4 mmHg, *p* = 0.03). Other laboratory data on admission showed no significant differences between the two groups (Table 2).

**Table 1:** The differences of situation at cardiac arrest between the good and poor neurological prognosis groups

	**All (*n* = 39)**	**CPC 1, 2 (*n* = 8)**	**CPC 3, 4, 5 (*n* = 31)**	***p* value**
Age (years)	56.1 ± 13.1	46.8 ± 11.1	58.5 ± 12.6	0.03
Gender male *n*, (%)	33 (84.5)	5 (62.5)	28 (90.3)	0.09
Cause *n*, (%)				
Acute coronary syndrome	19 (48.7)	7 (87.5)	12 (38.7)	0.03
Cardiomyopathy	14 (35.9)	0 (0)	14 (45.2)	
Arrhythmia	4 (10.3)	0 (0)	4 (12.9)	
Unknown	2 (5.1)	1 (12.5)	1 (3.2)	
Time from emergency call to hospital arrival (min)	30.6 ± 13.2	25.0 ± 9.8	32.1 ± 13.7	0.11
Age *n*, (%)				
Under 75	35 (89.7)	8 (100)	27 (87.1)	0.56
Over 75	4 (10.3)	0 (0)	4 (10.3)	
Shockable rhythm *n*, (%)				
Yes	34 (87.1)	7 (87.5)	27 (87.1)	1.00
No	5 (12.8)	1 (12.5)	4 (10.3)	
Cardiac arrest on arrival				
Yes	39 (100)	8 (100)	31 (100)	-
No	0 (0)	0 (0)	0 (0)	
Time from emergency call to hospital arrival within 40 min *n*, (%)				
Yes	31 (79.5)	8 (100)	23 (74.2)	0.17
No	8 (20.5)	0 (0)	8 (25.8)	
Witness *n*, (%)				
Yes	36 (92.3)	8 (100)	28 (90.3)	1.00
No	3 (7.7)	0 (0)	3 (9.7)	
Bystander CPR *n*, (%)				
Yes	26 (66.7)	8 (100)	18 (58.1)	0.04
No	13 (33.3)	0 (0)	13 (41.9)

**Table 2:** The other characteristics of study patients

	**All (*n* = 39)**	**CPC 1, 2 (*n* = 8)**	**CPC 3, 4, 5 (*n* = 31)**	***p* value**
Body mass index (kg/m^2^)	26.6 ± 4.5	26.0 ± 4.0	26.8 ± 4.7	0.69
Left ventricular ejection fraction (%)	17.9 ± 12.4	19.2 ± 7.4	17.6 ± 13.3	0.79
Diabetes mellitus (*n*, %)	7 (17.9)	3 (37.5)	4 (12.9)	0.29
Dyslipidemia (*n*, %)	31 (79.5)	8 (100)	23 (74.2)	1.0
Hypertension (*n*, %)	12 (30.8)	3 (37.5)	9 (29.0)	0.24
Smoking (current smoker) (*n*, %)	11 (28.2)	5 (62.5)	6 (19.4)	0.38
Laboratory data				
Total cholesterol (mg/dL)	150.9 ± 58.1	111.8 ± 51.2	161.2 ± 56.5	0.09
Triglycerides (mg/dL)	112.8 ± 71.1	110.9 ± 57.5	113.4 ± 75.8	0.46
HDL-C (mg/dL)	33.5 ± 12.2	31.0 ± 10.3	34.2 ± 12.8	0.48
LDL-C (mg/dL)	92.7 ± 38.2	71.9 ± 33.9	99.2 ± 37.8	0.08
eGFR (ml/min)	46.6 ± 17.1	51.1 ± 22.7	45.5 ± 15.5	0.52
Creatine kinase (IU/L)	420 ± 1158	234 ± 291	468 ± 1291	0.36
Albumin (g/dL)	3.35 ± 0.54	3.08 ± 0.75	3.43 ± 0.46	0.24
Potassium concentration (mM/L)	4.5 ± 0.8	4.2 ± 0.7	4.6 ± 0.8	0.28
D-dimar (μg/ml)	27.7 ± 48.9	19.8 ± 29.8	29.8 ± 53.2	0.56
Soluble fibrin (μg/ml)	33.8 ± 34.5	21.7 ± 22.4	36.8 ± 37.2	0.35
Brain natriuretic peptide (pg/mL)	292 ± 683	675 ± 1407	187 ± 228	0.36
Blood gas analysis				
Potential of hydrogen	6.94 ± 0.16	6.99 ± 0.18	6.93 ± 0.15	0.38
Lactate (mg/dL)	102.9 ± 32.8	107.1 ± 40.2	101.9 ± 31.2	0.74
Bicarbonate (mmol/L)	16.3 ± 4.5	13.4 ± 4.1	17.1 ± 4.1	0.04
PCO_2_ (mmHg)	79.3 ± 28.6	58.3 ± 26.4	84.7 ± 27.0	0.03

Our results had demonstrated a significant association between the presence of bystander CPR and better neurological prognosis, thus patients were divided into two groups: those who had bystander CPR and those who did not (Table 3). No significant differences were found in the age, gender, and shockable rhythm categories. In the group that had bystander CPR, the time from CA to hospital arrival was significantly faster. The levels of pH, lactate, bicarbonate, and PCO_2_ on admission did not show significant differences in the presence or absence of bystander CPR.

**Table 3:** The differences of patient's characteristics between the presence or absence of bystander CPR

	**All (*n* = 39)**	**Bystander CPR (+) (*n* = 26)**	**Bystander CPR (-) (*n* = 13)**	***p* value**
Age (years)	56.1 ± 13.1	54.3 ± 14.0	59.6 ± 10.6	0.20
Gender male *n*, (%)	33 (84.5)	21 (81.0)	12 (92.3)	0.64
Cause *n*, (%)				
Acute coronary syndrome	19 (48.7)	12 (46.2)	7 (53.8)	0.50
Arrhythmia	4 (10.3)	2 (7.7)	2 (15.4)	
Cardiomyopathy	14 (35.9)	10 (38.5)	4 (30.8)	
Unknown	2 (5.1)	2 (7.7)	0 (0)	
Time from emergency call to hospital arrival (min)	30.6 ± 13.2	26.8 ± 13.0	38.2 ± 10.3	< 0.01
Shockable rhythm *n*, (%)				
Yes	35 (89.7)	22 (84.6)	13 (100)	0.28
No	4 (10.3)	4 (15.4)	0 (0)	
CPC *n*, (%)				
1, 2	8 (20.5)	8 (30.8)	0 (0)	0.04
3, 4, 5	31 (79.5)	18 (69.2)	13 (100)	
Blood gas analysis				
Potential of hydrogen	6.94 ± 0.15	6.97 ± 0.15	6.89 ± 0.17	0.16
Lactate (mg/dl)	102.9 ± 32.8	101.6 ± 27.7	105.6 ± 42.3	0.76
Bicarbonate (mmol/L)	16.3 ± 4.5	16.1 ± 4.9	16.7 ± 3.9	0.70
PCO_2_ (mmHg)	79.3 ± 28.6	73.3 ± 25.2	91.2 ± 32.2	0.10

**Fig. 1:**
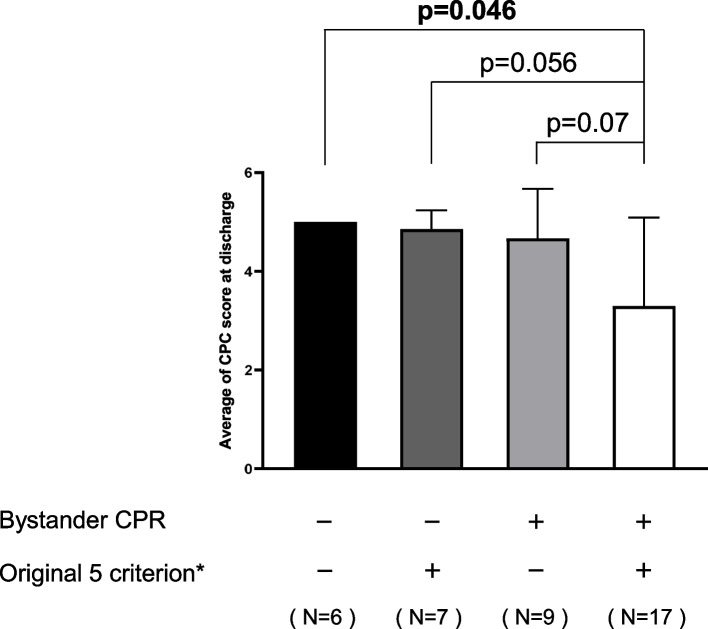
The average of CPC score at discharge was observed in combination with the presence of bystander CPR and all five original criteria (age 75 years or younger, cardiac arrest on arrival at hospital, within 40 min from CA to hospital arrival, shockable rhythm, and good ADL)

The mean CPC at discharge was compared based on the combination with the presence of bystander CPR and all five original criteria (age 75 years or younger, CA on arrival at the hospital, within 40 min from CA to hospital arrival, shockable rhythm, and ADL independence). As shown in Fig. 1, patients who received bystander CPR and met all original five criteria showed significantly better neurological prognosis compared to patients who did not receive bystander CPR and did not meet some of the original five criteria (3.3 ± 1.8 vs. 5.0 ± 0.0, *p* = 0.046). Cases with no bystander CPR revealed no significant differences in CPC levels between patients who met all five criteria or those who did not (4.9 ± 0.4 vs. 5.0 ± 0.0, not significant). In patients who met the five original criteria, the presence of bystander CPR tended to contribute to better neurological prognosis (3.3 ± 1.8 vs. 4.9 ± 0.4, *p* = 0.056).

## Discussion

No randomized controlled trials have been conducted on the use of V-A ECMO for CA; however, four observational studies since 2015 with > 100 cases of V-A ECMO revealed different results, without reliable V-A ECMO results [7–11]. This is mainly due to the differences in the implementation criteria and methods of V-A ECMO. The International Liaison Committee on Resuscitation guidelines unclearly state the criteria for V-A ECMO introduction; the enrollment criteria of a prospective study from another center (SAVE-J study) reported from Japan in 2014 include (1) shockable rhythm, (2) CA on arrival, (3) within 45 min from CA to hospital arrival, and (4) no return of spontaneous circulation after 15 min of CPR [12]. Considering that this study showed the usefulness of V-A ECMO, it is often used as a tentative criteria in Japan. However, certain appropriate implementation criteria are needed to guide the effectiveness of V-A ECMO.

The presence of bystander CPR affects the neurological prognosis of patients who had CA. In patients who survive out-of-hospital CA, bystander CPR has been associated with the risk of post-resuscitation brain injury and death from any cause. Currently, no consensus has been made on bystander CPR contribution to a favorable neurological prognosis in V-A ECMO cases [13]. However, our study showed that bystander CPR may also contribute to the neurological prognosis of V-A ECMO cases. In the group with bystander CPR, the time from CA to hospital arrival was significantly faster, suggesting that bystanders with knowledge of CPR were able to do emergency calls smoothly. This earlier arrival may contribute to improving the neurological prognosis. Inadequate tissue oxygenation can lead to anaerobic metabolism and metabolic acidosis, but cardiac massage is known to excrete arrhythmogenic substances, such as lactate and peri-myocardial potassium. Many studies reported that an increased pH and a decreased lactate are associated with a better neurological prognosis [14, 15], which also improves systemic and pulmonary blood flow and decreases PCO_2_, increasing the likelihood of ROSC [16]. No significant differences were found in pH, lactate, serum potassium, and PCO_2_ levels on admission, thus checking the blood data immediately after bystander CPR may be necessary rather than after hospital arrival.

This single-center retrospective observational study included a small number of cases as 39. Randomly assigning the presence or absence of bystander CPR was impossible. PCI, IABP, and TTM may have contributed to outcome improvement in participants, but the adjustment of these factors was difficult due to the small number of cases.

## Conclusion

The presence or absence of bystander CPR was associated with neurological prognosis in patients with V-A ECMO at our hospital. In the future, introducing the presence or absence of bystander CPR as a new criterion for introducing V-A ECMO was suggested, as predicting the neurological prognosis of CA cases and selecting appropriate cases may be possible.

## Data Availability

All data generated or analyzed during this study are included in this published article.
